# Designing Clinical Trials for Anti-Inflammatory Therapies in Cystic Fibrosis

**DOI:** 10.3389/fphar.2020.576293

**Published:** 2020-09-11

**Authors:** Lucy Perrem, Felix Ratjen

**Affiliations:** ^1^ Division of Respiratory Medicine, The Hospital for Sick Children, Toronto, ON, Canada; ^2^ Department of Paediatrics, University of Toronto, Toronto, ON, Canada; ^3^ Translational Medicine Program, SickKids Research Institute, Toronto, ON, Canada

**Keywords:** anti-inflammatories, cystic fibrosis, clinical trials, lung, airway inflammation

## Abstract

The inflammatory response in the CF airway begins early in the disease process and becomes persistent through life in most patients. Inflammation, which is predominantly neutrophilic, worsens airway obstruction and plays a critical role in the development of structural lung damage. While cystic fibrosis transmembrane regulator modulators will likely have a dramatic impact on the trajectory of CF lung disease over the coming years, addressing other important aspects of lung disease such as inflammation will nevertheless remain a priority. Considering the central role of neutrophils and their products in the inflammatory response, potential therapies should ultimately affect neutrophils and their products. The ideal anti-inflammatory therapy would exert a dual effect on the pro-inflammatory and pro-resolution arms of the inflammatory cascade, both of which contribute to dysregulated inflammation in CF. This review outlines the key factors to be considered in the design of clinical trials evaluating anti-inflammatory therapies in CF. Important lessons have been learned from previous clinical trials in this area and choosing the right efficacy endpoints is key to the success of any anti-inflammatory drug development program. Identifying and validating non-invasive biomarkers, novel imaging techniques and sensitive lung function tests capable of monitoring disease activity and therapeutic response are important areas of research and will be useful for the design of future anti-inflammatory drug trials.

## Introduction

Airway inflammation plays a critical role in the development of bronchiectasis in cystic fibrosis (CF) and contributes to the progressive decline in lung function (Pillarisetti et al., 2011; Sagel et al., 2012; Sly et al., 2013; Nichols and Chmiel, 2015). Inflammation typically beings early in the disease process and becomes persistent in most patients (Pillarisetti et al., 2011; Sagel et al., 2012; Sly et al., 2013; Nichols and Chmiel, 2015).

The inflammatory process in CF is predominantly driven by neutrophils (Cohen-Cymberknoh et al., 2013). Neutrophils are recruited into the airway in response to infection and then kill bacteria by releasing their contents including peroxidases and proteases (Watt et al., 2005). A key feature of inflammation in CF is that the response is excessive relative to the burden of infection due to a combination of increased influx and decreased clearance of pathogens and inflammatory cells (Nichols and Chmiel, 2015). Normally, once the intracellular contents are released, neutrophils undergo apoptosis by alveolar macrophages. However, in the CF airway, neutrophils undergo necrosis rather than apoptosis and this results in the release of damaging intracellular contents and chemoattractants and fuels further neutrophil influx (Watt et al., 2005; Cohen-Cymberknoh et al., 2016). The release of massive quantities of neutrophil elastase overwhelms the endogenous anti-proteases, such as anti-1 antitrypsin, needed to neutralize proteolytic activity, and this ultimately leads to the destruction of lung tissue (Birrer et al., 1994). The actions of neutrophil elastase also work to make the neutrophils in CF airways less effective at killing bacteria, partly due to cleavage of immunoglobulins and complement (McElvaney, 2016). This becomes a vicious cycle of neutrophilic inflammation, protease release, and oxidative stress which leads to tissue destruction and fibrosis of the lungs (Cohen-Cymberknoh et al., 2013).

Cystic fibrosis transmembrane regulator (CFTR) modulators are a new class of therapy that targets the basic genetic defect and could dramatically change the landscape of CF care. These drugs are potentially efficacious for approximately 90% of the CF population (Bell et al., 2020). However, despite the impressive improvements in lung function observed with ivacaftor in CF patients with gating mutations (Whiting et al., 2014), there is limited evidence that CFTR modulators impact inflammation. In a multicenter prospective cohort study in the post-approval setting, Rowe and co-workers found that patients on ivacaftor experienced significant improvements in forced expiratory volume in 1 second (FEV_1_) and reductions in sweat chloride, but there were no significant changes in any sputum inflammatory markers (Rowe et al., 2014). In contrast, Hisert and co-workers evaluated the impact of ivacaftor on airway inflammation over a longer period of time and reported a significant reduction in sputum inflammatory markers including neutrophil elastase, IL-8 and IL-1b (Rowe et al., 2014). However, even in this study, most patients had ongoing chronic infection and inflammation, albeit at a lower level. A large observational study designed to evaluate the effects of the triple combination CFTR modulator (elexacaftor/tezacaftor/ivacaftor) may be able to more definitively establish how this class of drug impacts airway inflammation (Block et al., 2006).

Considering that the clinical response to CFTR modulators is variable, they are not universally available and, if bronchiectasis is already established, chronic infection and inflammation persist and lung function continues to decline (Sawicki et al., 2015), developing new therapies that target other aspects of CF lung disease remains a priority (Perrem and Ratjen, 2019). The purpose of this review is to outline key aspects to be considered in the design of clinical trials evaluating the efficacy of anti-inflammatory therapies.

## What Is the Ideal Inflammatory Pathway to Target?

The complexity of the inflammatory process in CF provides multiple potential targets for intervention. Due to the central role of neutrophils in the inflammatory process, effective anti-inflammatory therapies must target neutrophils or their products (Torphy et al., 2015).

The CF airway contains a broad spectrum of pro-inflammatory mediators, such as TNF-alpha, IL-1beta, IL-6, IL-8, IL-17, IL-33. GM-CSF, G-CSF, and HMGB-1 (Nichols and Chmiel, 2015). The downregulation of inflammation is also defective, with many studies demonstrating a deficiency in counter-regulatory molecules such as IL-10 and lipoxin-A4 (LXA4). The multiplicity of inflammatory pathways and inherent redundancy in the process makes it challenging to target specific components, both the pro-inflammatory and pro-resolving pathways offer potential targets for therapeutic interventions.

Longitudinal data from the Australian Respiratory Early Surveillance Team for Cystic Fibrosis (*AREST* CF) surveillance program demonstrated that free neutrophil elastase activity in bronchoalveolar lavage (BAL) fluid at 3 months of age was associated with bronchiectasis on computed tomography (CT) scan at 1 and 3 years of age (Sly et al., 2013). While it is unclear whether targeting a specific mediator of inflammation will be successful, given the intense burden of neutrophil elastase in CF and the predictive value of this biomarker, neutrophil elastase is a key target for anti-inflammatory therapy. Studies investigating alpha-1 antitrypsin therapy as a way to restore the protease-antiprotease balance in the CF airway have shown an improvement in neutrophil function, a reduction in inflammation, and augmentation on bacterial clearance (McElvaney et al., 1991; Birrer et al., 1994; Griese et al., 2007; McElvaney, 2016). This provides proof of concept that this approach could be successful in future clinical trials.

## Lessons Learned From Previous Trials

Previous studies investigating anti-inflammatory therapies have demonstrated the importance of choosing the right target and the right dose of active drug.

The major concern in undertaking trials with anti-inflammatory agents in CF is the potential that suppressing the inflammatory response in a chronically infected airway will impair host defense and exacerbate infections (Torphy et al., 2015). We learned these lessons from a large phase II clinical trial of an LTB4 receptor antagonist (BIIL 284 BS). The CF inflammatory response is mediated in part by LTB4, a key modulator of inflammation that attracts and activates neutrophils in the airway. Preclinical studies suggested that LTB4-receptor inhibition could have positive clinical effects (Konstan et al., 1993) but the phase II BIL 284BS trial was terminated early because of increased pulmonary exacerbations in the adults treated with the study drug, combined with evidence of decreased pulmonary function and increased circulating neutrophils (Konstan et al., 2014). To understand the mechanisms underlying these negative results, subsequent studies in mice found that lower doses of the drug attenuated the inflammatory response without increasing infection, but high doses, like the dose used in the clinical trial, overly suppressed the inflammatory response and were associated with increased bacterial colony counts (Döring et al., 2014). Therefore, future trials evaluating anti-inflammatory drugs in the clinical trial setting should first have generated sufficient information about the possible harms of the drug. Preclinical studies or data available from studies investigating the same drug in other patient populations are essential to ensure a drug is safe for clinical trials in CF. **Table 1** describes how the study design and outcome measure will vary with the development phase when investigating anti-inflammatory therapies in cystic fibrosis.

**Table 1:** The study design and outcome measure will vary with the development phase of an anti-inflammatory therapy.

Phase of development	Population	Duration	Outcome measures	Example
**Preclinical study**	Animal models In vitro assays	Days	**Safety** Lack of negative effect on airway infection	CFTR-deficient mouse model to investigate infection susceptibilities associated with study drug (Bonfield, 2020)
**Phase I**	Healthy volunteers or Non-CF patients	≤3 months	**Safety and pharmacokinetics**	Phase 1 study of acebilistat in CF patients and healthy volunteers (Elborn et al., 2017)
**Phase IIa**	CF adults	8–12 weeks	**Biomarkers** **e.g. sputum NE, IL-6, IL-8** **serum CRP, calprotectin** Trends in a reduction in PEx *New imaging techniques e.g. MRI, PET-CT* *Functional tests e.g. MBW*	Phase II randomized placebo-controlled trial of acebilustat 48-week study Primary endpoint: change in FEV1pp and safety outcomes Secondary outcomes: PEx rate, time to first PEx and biomarkers of inflammation (Elborn et al., 2018)
**Phase IIb/III**	CF adolescents and adults	≥6 months	**PEx rate** FEV1 decline *Evolution of bronchiectasis on CT*	Phase IIb placebo-controlled trial of lenabasum in cystic fibrosis. 28-week study Primary outcome: PEx rate Secondary outcomes: time to first PEx; PEx rate using secondary PEx definition; change in CFQ-R Respiratory domain score; Change in FEV1pp; Adverse events (Trial to Evaluate Efficacy and Safety of Lenabasum in Cystic Fibrosis—Full Text View—ClinicalTrials.gov)
**Alternative study design**	CF adolescents and adults	Treatment during PEx	**FEV1 change with treatment** **FEV1 recovery post treatment** Biomarkers	Randomized placebo-controlled trial Of prednisone in cystic fibrosis PEx Primary outcome the proportion of subjects who achieve >90% of baseline FEV1% predicted at day 14 of IV antibiotic treatment (Prednisone in Cystic Fibrosis Pulmonary Exacerbations - Full Text View - ClinicalTrials.gov)

NE, neutrophil elastase; CRP, C-reactive protein; CFQ-R, Cystic Fibrosis Questionnaire- Revised; MRI, magnetic resonance imaging; PET-CT positron emission tomography – computed tomography; PEx; pulmonary exacerbation; FEV1pp, FEV_1_ percent predicted; MBW, multiple breath washout.

Key outcome measures in bold, potential future outcome measures in italics.

## Role of Biomarkers

Given the important role of inflammation in CF lung disease, identifying biomarkers capable of monitoring disease activity or therapeutic response would be very useful (Tiddens et al., 2015). In clinical drug development, biomarkers can be used in early phase studies to demonstrate the biological safety and efficacy of new therapies, confirm the mechanism of action and inform dose selection (Muhlebach et al., 2016). Biomarkers can also be useful to compare results from preclinical and clinical studies (Torphy et al., 2015; Muhlebach et al., 2016). The relatively poor success rate in developing new anti-inflammatory therapies can, in part, be attributed to a lack of accurate, reproducible, noninvasive biomarkers that reflect the anti-inflammatory process (Martinez et al., 2011).

### Lung-Derived Inflammatory Biomarkers

Bronchoalveolar lavage (BAL) is considered the gold standard for quantifying airway inflammation in the CF (Tiddens et al., 2015). However, the use of BAL longitudinally is limited by the invasiveness of the procedure. Sputum is an alternative way of obtaining material directly from the site of inflammation. For younger subjects or those with mild lung disease who cannot expectorate, sputum induction improves sample acquisition and biomarker measurements and are reasonably comparable with expectorated sputum (Sagel et al., 2001; Zemanick et al., 2015). However, both spontaneously expectorated and induced sputum are variable, making it difficult to track patients over time (Sagel et al., 2007; Chmiel et al., 2015).

The utility of sputum biomarkers of inflammation in CF clinical trials has been previously reviewed in detail, with findings from multiple studies supporting the association between pro-inflammatory cytokines and disease status in CF subjects (Sagel et al., 2007; Tiddens et al., 2015; Muhlebach et al., 2016). A secondary analysis of data from four randomized controlled trials, which included a diverse CF population, demonstrated that free neutrophil elastase and IL-8 were negatively correlated with FEV1. Neutrophil elastase, a key mediator of lung damage, was the inflammatory marker with the strongest relationship with FEV_1_ (Mayer-Hamblett et al., 2007), correlating with FEV_1_ both cross-sectionally and longitudinally. As neutrophil elastase correlates with bronchiectasis (DeBoer et al., 2014), tracks with and is predictive of future lung function decline (Mayer-Hamblett et al., 2007; Sagel et al., 2012), relates to treatment response and predicts time to next exacerbation (Ordoñez et al., 2003; Waters et al., 2015), it is currently considered the most informative sputum biomarker to monitor CF lung disease.

Another sputum biomarker with supportive longitudinal data is high-mobility group box-1 protein (HMGB-1); this inflammatory marker predicted subsequent pulmonary exacerbations and survival during 7 years of follow-up (Liou et al., 2001).

Despite these strong observational data, it is still not clear what magnitude of biomarker change that could be considered clinically meaningful in the interventional setting. Furthermore, for biomarkers to convincingly reflect the treatment effect of an investigational drug, multiple biomarkers should improve rather than just a single one. Chmiel and colleagues designed a placebo-controlled randomized trial to investigate whether sputum biomarkers could be used to screen candidate anti-inflammatory therapies over a short period of time (Chmiel et al., 2015). Results from a screening study such as this could provide a go-no-go decision on whether to proceed with a phase 2 trial. Given than high-dose ibuprofen is the only anti-inflammatory drug recommended for use in CF (Mogayzel et al., 2013), it was chosen to test this hypothesis. Ibuprofen is a non-steroidal anti-inflammatory drug that affects the cyclo-oxygenase pathway and results in the inhibition of prostaglandin synthesis and, at high doses, is associated with a reduction in neutrophil migration into the lung (Konstan et al., 2003). However, in this proof of concept study, there was no significant change in key inflammatory markers, including neutrophil elastase, over a 28-day trial period. It is conceivable that a longer trial is required to see a decrease in inflammatory markers in a chronically infected airway; alternatively, ibuprofen may not have been the ideal drug to test the hypothesis.

### Blood-Based Inflammatory Markers

Blood-based markers are relatively non-invasive, are easily standardized and can be obtained from subjects of any age and disease severity. Although the data linking blood-based inflammatory markers to clinical outcomes is less extensive than for sputum and BAL markers, systemic inflammatory biomarkers correlate with important clinical events including pulmonary exacerbations and lung function decline (Proesmans et al., 2011; Shoki et al., 2013; Reid et al., 2015; Quon et al., 2016).

A clinical trial of school-age children with CF uninfected with *Pseudomonas aeruginosa* investigated the responsiveness of a panel of systemic inflammatory markers to treatment with azithromycin (Ratjen et al., 2012). Azithromycin, a macrolide antibiotic, is presumed to exert its proinflammatory effect in the CF airway by reducing proinflammatory cytokine production by cells such as neutrophils, monocytes, and bronchial epithelial cells; although its precise mechanism of action remains unclear (Parnham et al., 2005). The trial by Ratjen and co-workers demonstrated that circulating neutrophil counts, C-reactive protein, serum amyloid A, and calprotectin all significantly reduced within 28-days of treatment. Furthermore, reductions in these inflammatory markers were correlated with improvements in lung function and weight gain, providing indirect evidence that these changes were associated with clinically meaningful outcomes. A secondary analysis of this study data showed that early changes in serum calprotectin levels after the first 28 days of azithromycin treatment were predictive of pulmonary exacerbation risk by day 168 (Dong et al., 2019). This demonstrates that early changes in biomarkers have the potential to predict meaningful longer-term outcomes and could be useful outcome measures in interventional trials.

## Capturing the Effect on Lung Disease

### Spirometry

Reduced FEV_1_, derived from spirometry, is strongly linked with increased morbidity and mortality and is, therefore, a key outcome measure in CF clinical studies (Kerem et al., 1992). Unlike drugs targeting other aspects of CF lung disease (e.g., CFTR modulators and mucoactive drugs) which have shown improvements in FEV_1_ within 14 to 28 days (Fuchs et al., 1994; Ramsey et al., 2011), trials evaluating anti-inflammatory drugs have not reported immediate effects on lung function (Perrem and Ratjen, 2019). However, failure to show short-term improvements in expiratory flows airway resistance does not necessarily predict long-term benefits in lung function decline. This was demonstrated by trials investigating ibuprofen, a non-steroidal anti-inflammatory drug that reduces neutrophil influx into the lung a at higher dose (Mogayzel et al., 2013). High dose ibuprofen does not improve FEV_1_, but data from two prospective clinical trials that included 226 participants, showed that the use of ibuprofen twice daily slows the decline of FEV_1_ (Konstan et al., 2007; Lands et al., 2007; Lands and Stanojevic, 2019). A recent observational study also demonstrated that this beneficial effect of high-dose ibuprofen translates to improved survival (Konstan et al., 2018). Ibuprofen, therefore, provides proof of concept that targeting inflammation might improve outcomes for patients with CF, but that short-term benefits in lung function may not be seen. However, this does not exclude the possibility that a more potent anti-inflammatory compound could potentially achieve this. Interventional studies using lung function decline as an endpoint would need to be conducted over multiple years with large sample sizes size and few drug development programs would be willing to take this route. This highlights the need to identify sensitive biomarkers that can more rapidly screen candidate drugs and be used as surrogate endpoints in phase II trials reducing the failure of drugs in phase III.

A *post hoc* analysis of data from the ibuprofen trial from Konstan et al. showed a slower rate of annual decline in lung function in the ibuprofen group in younger children than in those 13 years and older (Konstan et al., 2018). The findings were consistent for all lung function outcomes (FEV_1_, FVC, and FEF 25%–75%) and suggest that ibuprofen is more efficacious when used in individuals with mild CF lung disease. It is also possible that it is the trajectory of lung function and not just baseline FEV_1_ that influences response to therapy. Future trials should consider stratifying patients not only based on the severity of lung disease but also on the trajectory of lung function over time.

### Multiple Breath Washout Test

With more CF patients categorized as early lung disease with FEV_1_ in the normal range, there is an urgent need for more sensitive outcome measures (Tiddens et al., 2015). The lung clearance index (LCI), derived from the multiple breath washout (MBW) test, reflects ventilation inhomogeneity with higher values indicating more severe lung disease. LCI is a reliable, valid, and responsive functional test and is now an established outcome measure in interventional trials (Kent et al., 2014). LCI also correlates with markers of systemic inflammation(Horsley et al., 2013; O’Neill et al., 2018), including CRP and calprotectin, and with the extent of airway inflammation (Ramsey et al., 2017). The LCI has been shown to detect treatment effects to medications such as hypertonic saline in trials involving both preschool (Subbarao et al., 2013; Ratjen et al., 2019) and school-age CF subjects with preserved spirometry (Amin et al., 2010; Amin et al., 2011; Davies et al., 2013; Ratjen et al., 2017), where a change in FEV_1_ with treatment was not detected. The published treatment effects for LCI range from less than 1 units (0.6 for hypertonic saline, 0.9 units for dornase alfa) (Amin et al., 2011; Stanojevic and Ratjen, 2016) up to 2.2 units for ivacaftor in patients with CFTR gating mutations (Davies et al., 2013). LCI has not yet been incorporated into the design of a clinical trial for an anti-inflammatory therapy but the enhanced sensitivity to detect treatment effects compared to FEV_1_ may potentially facilitate anti-inflammatory trials in the future.

### Pulmonary Exacerbations

Pulmonary exacerbations are important clinical events in the disease process and directly contribute to the progression of lung disease (Sanders et al., 2010; Heltshe et al., 2016; Stanojevic et al., 2017; van Horck et al., 2018). Therefore, pulmonary exacerbations also serve as meaningful clinical efficacy endpoints in interventional trials. Multiple clinical trials investigating different classes of CF medications, such as CFTR modulators (Davies et al., 2013; Ratjen et al., 2018) dornase alfa (Fuchs et al., 1994), hypertonic saline (Elkins and Dentice, 2020), and tobramycin (Ramsey et al., 1999) have shown a reduction in pulmonary exacerbations compared with placebo. Given the role of inflammation in pulmonary exacerbations, targeting the inflammatory process should logically result in a reduction in pulmonary exacerbations but this has not yet been proven in phase III trials. Furthermore, given the improved overall state of health in CF, powering a study to pulmonary exacerbation endpoints, particularly in patients with mild lung disease, require large numbers of subjects, and longer follow-up times to demonstrate a treatment effect. With respect to pulmonary exacerbations, it is unclear as to what is the best definition and surrogate outcome measure — risk, frequency, or time to the next pulmonary exacerbation event. Studies using pulmonary exacerbations as an endpoint could reduce the number of patients required by limiting recruitment to individuals with a recent history of exacerbations as this has predictive value for future events (Block et al., 2006). This concept has been implemented in the design of a phase 2 study investigating lenabasum (Chmiel and Elborn); a drug that acts as a selective agonist of the cannabinoid receptor on type 2 immune cells and exerts anti-inflammatory and pro-resolution effects without suppressing the immune system (Motwani et al., 2018).

The ideal timing to initiate anti-inflammatory therapies is unclear, whether they would be most efficacious if initiated when a patient is clinically stable or during a pulmonary exacerbation when the inflammatory process is at its peak. A pilot randomized controlled trial investigating the short-term use of oral prednisone in CF patients presenting with pulmonary exacerbations demonstrated a modest improvement in lung function (Dovey et al., 2007). The PIPE study is an ongoing multisite randomized placebo-controlled trial that is investigating the efficacy of oral prednisone as an adjunctive therapy during pulmonary exacerbations. If successful, this study design could provide a model for evaluating other anti-inflammatory therapies in the future (Prednisone in Cystic Fibrosis Pulmonary Exacerbations - Full Text View - ClinicalTrials.gov).

### Other Outcome Measures

Various types of imaging techniques are now available to determine the presence and extent of lung disease in patients with CF, including CT and chest magnetic resonance imaging (MRI) (Tiddens et al., 2015). Studies have demonstrated that infection, inflammation, and abnormal chest CT findings are already present in a significant proportion of asymptomatic infants with CF at 3-months of age (Sly et al., 2009) and that these early structural changes are progressive (Mott et al., 2012). On the other end of the spectrum, in CF patients screened for lung transplantation, those with a higher volume of infection/inflammation-like changes were shown to have a higher risk of dying on the waiting list (Loeve et al., 2009). Furthermore, individuals with more extensive structural lung disease on CT experience more pulmonary exacerbations (Brody et al., 2005; Loeve et al., 2011).

While chest CT scans are sensitive at detecting structural changes, the evolution of these changes over time is slow, with bronchiectasis deteriorating at about 1.5% per year on serial CT scans in CF patients(De Jong et al., 2006). Therefore, trials using the development of bronchiectasis on CT as the primary endpoint would take multiple years and/or large numbers of subjects (De Jong et al., 2006; Owens et al., 2011).

Pulmonary MRI can now provide high‐resolution images that are sensitive to early disease and specific to inflammation in cystic fibrosis (CF) lung disease (Amin and Ratjen, 2008; Tiddens et al., 2015; Ciet et al., 2017; Woods et al., 2019). Unlike CT, MRI does not use ionizing radiation, and this is particularly advantageous in children and when scans need to be repeated within a relatively short time period (Amin and Ratjen, 2008; Tiddens et al., 2015). MRI techniques can track changes in lung function longitudinally and quantify treatment response (Rayment et al., 2018; Santyr et al., 2019; Woods et al., 2019). Proton density and T_1_/T_2_ contrast images can be obtained within a single breath‐hold, providing a depiction of structural abnormalities and active inflammation. Hyperpolarized‐gas MRI, increasingly using ^129^Xe, is now becoming more widespread and has been demonstrated to have high sensitivity to early airway obstruction in CF and could have utility as an endpoint in future clinical trials, particularly in the acute setting of pulmonary exacerbations.

Another promising non-invasive imaging technique that provides information about the level of inflammation is positron emission tomography (PET) with [18]fluorodeoxyglucose ([18]FDG; FDG-PET). 18F-FDG is taken up by activated neutrophils, macrophages, and lymphocytes so in contrast to other imaging techniques, FDG-PET scans assess CF airway inflammation directly. Several observational studies have shown that 18F-FDG uptake can determine the location and intensity of pulmonary inflammation and, combined with CT, PET can be used to assess anatomy and structures. (Labiris et al., 2003; Chen et al., 2006; Klein et al., 2009; Amin et al., 2012). A study by Amin and co-workers, demonstrated that FDG PET/CT depicts changes in inflammation in the lung after intravenous antibiotics for a pulmonary exacerbation, with the PET signal correlating with the burden of sputum neutrophils (Amin et al., 2012). Data from this study support the utility of FDG PET/CT as an outcome measure in treatment studies, although it is not yet a validated outcome for use in interventional trials.

## CFTR Modulators and Anti-Inflammatory Therapies

Time will tell how increasing CFTR modulator access will affect future anti-inflammatory therapeutic development. The positive effects of CFTR modulators on the progression of CF lung disease are undoubtedly positive but this will make the issues of endpoints for anti-inflammatory trials event more challenging.

Acebilustat is an inhibitor of leukotriene A4 hydrolase (LTA4H), an enzyme that catalyzes the rate-limiting step in the formation of leukotriene B4 (LTB4), a potent chemoattractant and activator of inflammatory immune cells including neutrophils (Rao et al., 2010). This anti-inflammatory drug is also proposed to work by shunting substrates down the metabolic pathway to produce the pro-resolving mediator LXA4 (Tobin et al., 2010). In the Acebilustat phase IIb trial, the observed reduction in pulmonary exacerbations during the 48-week study period was also observed in the subgroup of patients taking CFTR modulators (Griese et al., 2007). This suggests that patients taking modulators may receive additional benefit from anti-inflammatory drugs. It would be important for future trials to take this into account and stratify groups based on whether subjects are taking CFTR modulators.

## Conclusion

Designing trials of anti-inflammatory therapies in CF faces specific challenges different from other drug development programs. Demonstrating safety data from preclinical studies, choosing appropriate and realistic efficacy endpoints and integrating sensitive imaging and lung function outcomes into future trials are important considerations. These measures will increase the likelihood that potentially efficacious therapies are not abandoned prematurely and that efficacy can be conclusively demonstrated in studies that can be completed in reasonable time frames.

## Author Contributions

Both authors conceptualized the content of the review. LP wrote the first draft and FR reviewed and edited the final manuscript.

## Conflict of Interest

The authors declare that the research was conducted in the absence of any commercial or financial relationships that could be construed as a potential conflict of interest.

## Abbreviations

CF, cystic fibrosis; CFTR, cystic fibrosis transmembrane regulator; NE, neutrophil elastase; MRI, magnetic resonance imaging; CT, computed tomography; PET, positron emission tomography; MBW, multiple breath washout; FEV1, forced expiratory volume in 1 s.
